# Does bipolar disorder cause posttraumatic growth? Relationship between psychological resistance in patients with bipolar disorder and caregivers

**DOI:** 10.1192/j.eurpsy.2021.512

**Published:** 2021-08-13

**Authors:** B. Kırşavoğlu, N. Akkişi Kumsar

**Affiliations:** Psychiatry, Istanbul Erenkoy Mental and Neurological Diseases Research Hospital, istanbul, Turkey

**Keywords:** Psychological Resistance, Posttraumatic Growth

## Abstract

**Introduction:**

Focusing on the negative changes experienced by both patients and caregivers associatively caregiving and experiencing chronic mental illnesses, there is an increasing interest in the phenomenon of development after traumatic experiences with high levels of stress. These changes are in line with the concept of posttraumatic growth.

**Objectives:**

In the study, posttraumatic growth and psychological resilience in bipolar patients and caregivers has been examined in the context of the variables that are claimed to be related to it.

**Methods:**

With the approval of ethics committee, 49 patients in euthymic period and caregivers, 49 healthy volunteers meeting the inclusion criterions, applied to Erenköy Mental and Neurological Diseases Training-Research Hospital outpatient clinics between July-December 2019 were included. While psychological resilience and posttraumatic growth scale were implemented to patients and caregivers only psychological resilience scale was applied to healthy volunteers. The relationship between posttraumatic growth and psychological resilience, patient and caregiver variables was examined through statistical methods.

**Results:**

Comparing with the patients and caregivers, respectively posttraumatic growth total scores were 57.7%-61.3% of the highest score obtained from the scale in the patients and caregivers. Considering the literature, patients and caregivers experienced moderate to high posttraumatic growth. Caregivers’ psychological resilience levels was higher than the other groups.

**Conclusions:**

The results of the study are in line with the findings that, negative life experiences positively contributes to individuals. Knowing the factors affecting posttraumatic growth can make contribution to approaching patients and caregivers in clinical practice.

